# Liquid Anaerobic Digestate as a Source of Nutrients for Lipid and Fatty Acid Accumulation by *Auxenochlorella Protothecoides*

**DOI:** 10.3390/molecules24193582

**Published:** 2019-10-04

**Authors:** Izabela Krzemińska, Marta Oleszek, Dariusz Wiącek

**Affiliations:** Institute of Agrophysics, Polish Academy of Sciences, Doświadczalna 4, 20-290 Lublin, Poland; m.oleszek@ipan.lublin.pl (M.O.); d.wiacek@ipan.lubin.pl (D.W.)

**Keywords:** biomass, microalgae, agriculture, wastewater, methane fermentation

## Abstract

In recent years, there has been growing interest in the biomass of unicellular algae as a source of valuable metabolites. The main limitations in the commercial application of microbial biomass are associated with the costs of production thereof. Maize silage is one of the main substrates used in biogas plants in Europe. The effects of sterilized agricultural liquid digestate (LD) from methane fermentation of maize silage on the growth rates, macro and micronutrient removal efficiency, lipid content, and fatty acid profile in *Auxenochlorella protothecoides* were investigated. The results indicate that *A. prothecoides* can proliferate and accumulate lipids with simultaneous reduction of nutrients in the 1:20 diluted liquid digestate. The rate of nitrogen and phosphorus removal from the liquid digestate was 79.45% and 78.4%, respectively. Cells growing in diluted liquid digestate exhibited the maximum lipid content, i.e., 44.65%. The fatty acid profile of *A. prothecoides* shows a decrease in the content of linolenic acid by 20.87% and an increase in oleic acid by 32.16% in the LD, compared with the control. The liquid digestate changed the content of monounsaturated fatty acids and polyunsaturated fatty acids. The cells of *A. protothecoides* growing in the liquid digestate were characterized by lower PUFA content and higher MUFA levels.

## 1. Introduction

Microalgae are currently regarded as a promising feedstock for the production of biofuels such as biodiesel, biogas, and hydrogen and valuable bioproducts such as pigments (chlorophylls, β-carotene, lutein, zeaxanthin, astaxanthin), lipids, carbohydrates, and vitamins [1,2]. However, the commercial use of microalgal biomass is still a big challenge, despite the extensive research. This is related to the high costs of cultivation, e.g., the cost of nutrients. Algal cultures require a considerable amount of nutrients, primarily nitrogen and phosphorus. The N-fertilizer consumption (kg/kg oil) in microalgal cultures is higher than in the cultivation of other oil-bearing crops [3]. To solve this problem, the use of industrial, agricultural, or municipal wastewaters, which are cheap sources of nutrients indispensable for algal growth, has been proposed [4].

*A. protothecoides* has been recognized as a promising microorganism to be used in biodiesel production [5]. In previous studies, *A. protothecoides* cultures were grown on waste substrates: anaerobically treated brewery wastewater [6], whey permeate [7], mixed waste substrate of brewer fermentation and crude glycerol waste [8], non-sterilized urban wastewater [9], hydrolyzate of waste activated sludge [10], concentrated municipal wastewater [11], and centrate wastewater [12]. 

Liquid digestate is an important by-product of the methane fermentation process. It is characterized by a high content of untreated nitrogen; therefore, it can be used as a potential waste material for cultivation of algae [13]. The present study assesses the ability of unicellular *A. protothecoides* to grow, synthesize lipids and fatty acids, and remove nutrients in liquid digestate from methane fermentation of maize silage. Previous studies on the application of liquid digestate as a substrate for algal growth were mainly conducted using liquid digestate derived from the process of anaerobic digestion of industrial waste and agriculture waste—mainly dairy manure, swine manure, livestock waste, and poultry litter [14]. However, there are no studies of the effect of liquid digestate from methane fermentation of maize silage on lipid content and changes in the fatty acid profile in oil-rich microalgal species such as *A. protothecoides*. The highest potential of maize silage as a substrate for biogas production resulted from the low nutrient demand of maize as well as its high water-use efficiency and high digestibility [15]. Maize silage exhibits favorable chemical properties important for biogas production, such as a low content of lignin and ash and a high content of simple sugars [16].

The aim of this study was to determine the possibility of using liquid digestate as a source of nutrients for oil-rich microalgae *A. protothecoides* and to assess the impact of liquid digestate on changes in metabolic processes. In this study, the content of lipids and changes in the fatty acid profile in *A*. *protothecoides* were evaluated in culture on liquid digestate to assess the potential for application of the lipids for biodiesel production. Additionally, the ability of *A. protothecoides* to remove macro and micronutrients was analysed.

The investigations presented in this paper demonstrate that sludge derived from the fermentation of maize silage can be a suitable substrate for microalgal growth and indicate the effect of liquid digestate on the production of *A. protothecoides*. This research is important, as maize silage is one of the widely applied feedstocks in biogas plants in Europe [17].

## 2. Results 

### 2.1. Effect of The Digestate on Growth Kinetics

**Figure 1:**
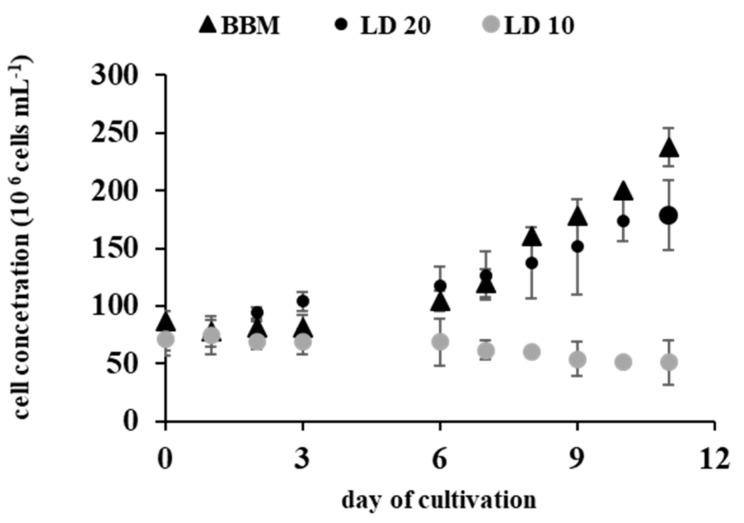
Growth curve of *A. protothecoides* (the results are presented as means of 9 measurements from *n* = 3; error bars represent standard deviation), the black triangles represent the number of cells in the control BBM medium; circles: the black circles represent the number of cells in the 1:20 liquid digestate (LD 20) and the grey circles represent the number of cells in the 1:10 liquid digestate (LD 10).

The experiments were conducted using digestate from methane fermentation of maize silage as a culture medium to assess the ability of *A. protothecoides* to grow and accumulate lipids in this substrate. *A. protothecoides* grew in a batch culture. The growth was determined at two dilutions of the liquid digestate: 10 and 20 relative to the control (BBM medium). Figure 1 shows changes in the number of cells in an 11-day experiment. 

The type of the medium significantly influenced the length of the growth phase and the rate of *A. protothecoides* growth. The growth of *A. protothcecoides* was inhibited in the 1:10 digestate dilutions; hence, further measurements and analyses were carried out only for a 1:20 digestate dilution. The liquid digestate (LD 20) stimulated the growth of *A. protothecoides* more efficiently than the control medium in the first phase of growth (days 0–6). As demonstrated by the growth curves (Figure 1), *A. protothecoides* exhibited a short lag phase, and exponential growth began on the second day of growth in the liquid digestate culture (LD 20) (Figure 1). 

In the control conditions, the growth curve showed two phases of growth, i.e., the lag phase and the exponential phase, but there was no stationary phase. *A*. *protothecoides* cells growing in the liquid digestate (LD 20) reached the stationary growth phase after the exponential phase, which may have been caused by nutrient availability and depletion. After 8 days of growth, the number of *A. protothcecoides* cells in the control culture increased and was higher than in LD 20. The highest number of cells, i.e., 2.30 × 10^8^ (cells mL^−1^) was detected on day 11 in the control conditions; this value was lower in the liquid digestate culture (1:20), i.e., 1.79 × 10^8^ (cells ml^−1^).

The effect of the liquid digestate on the specific growth rate was assessed on days 0–6 and 7–11. The comparison of the growth curves indicated that the use of LD 20 as a medium increased the cell concentration and enhanced the growth rate of *A. protohecoides* more efficiently than the control medium. The growth parameters of *A*. *protothecoides* are shown in Table 1. 

The maximum specific growth rate in the first phase of the culture (days 0–6) and the second culture phase (days 7–11) was obtained for cultures grown in the digestate dilutions (LD 20) and was µ = 0.412 d^−1^ and µ = 0.704 d^−1^, respectively. The minimum specific growth rate (µ = 0.302 d^−1^) was achieved in the control medium. The biomass doubling time was dependent on the medium used for the cultivation. *A. protothecoides* cultivated in LD 20 exhibited a shorter biomass doubling time than in the control conditions. 

### 2.2. Characterization of The Digestate and Removal of Macro- and Micronutrients by A. Protothecoides

The parameters of the digestate are presented in Table 2. The tested digestate is rich in ash and is characterized by relatively low content of volatile solids (VS). 

In comparison to BBM, the liquid digestate was characterized by a higher content of potassium (K), manganese (Mn), and iron (Fe) and a lower content of sodium (Na), zinc (Zn), molybdenum (Mo), magnesium (Mg), and copper (Cu) (Table 3). The liquid digestate did not contain cobalt (Co), which is a component of the BBM medium. The relative removal (in % of the initial concentration) from the liquid digestate was greater in the case of Na, Zn, Mg, Mo, and Cu, and the absolute Mo and Mg removal rate (in mg L^−1^) was higher compared to the BBM medium. 

**Figure 2:**
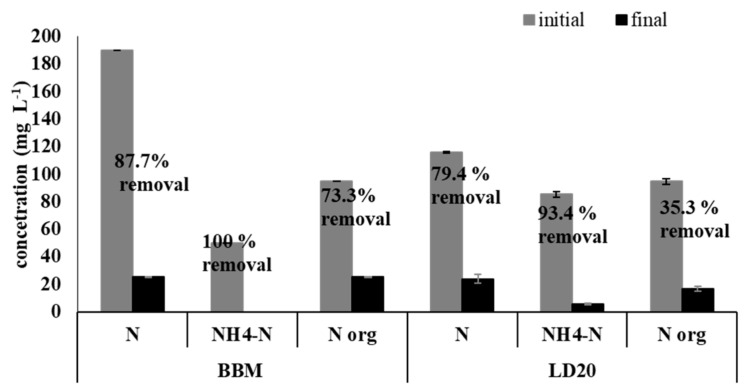
The initial and final concentration as well as the rate of removal of total nitrogen N, ammonia nitrogen NH_4_-N, and organic nitrogen Norg by *A*. *protothecoides* from BBM medium and liquid digestate (LD 20).

In the present study, LD 20 was characterized by an initial total nitrogen (N) concentration of 115.73 mg L^−1^, with organic nitrogen (Norg) accounting for 26.06 mg L^−1^ (Figure 2).

The initial N concentration in BBM with peptone was 189.55 mg L^−1^, which was a higher value than that in LD 20, and Norg represented 94.5 mg L^−1^. The removal of N reached 79.4% in the liquid digestate and 87.7% in the BBM medium. In turn, the level of Norg removal by *A. protothecoides* was 35.3% and 73.3% in the liquid digestate and BBM, respectively. Ammonia nitrogen (NH_4_-N) was the main source of nitrogen in the liquid digestate. The NH_4_-N content in the digestate was higher than in the control medium, i.e., 85.28 mg L^−1^ and 50.00 mg L^−1^ in LD 20 and BBM, respectively (Figure 2). 

The N-NO_3_^−^ content in LD 20 was low, i.e., 4.15 mg L^−1^ N-NO_3_^−^ in peptone-supplemented BBM, it was 45.05 mg L^−1^ N-NO_3_^−^ (Table 3). *A*. *protothecoides* assimilated N-NO_3_^−^ well, and a 71.1% reduction in the NO_3_-N level in the liquid digestate was shown after 11 cultivation days. Nitrite (N-NO_2_^−^) was detected in the liquid digestate in very small amounts (0.25 mg L^−1^).

The diluted liquid digestate was characterized by a low phosphorus content in comparison with the control medium. The initial concentration of phosphorus was 2.08 mg L^−1^ in LD 20 and 53.3 mg L^−1^ in BBM (Table 3).

**Figure 3:**
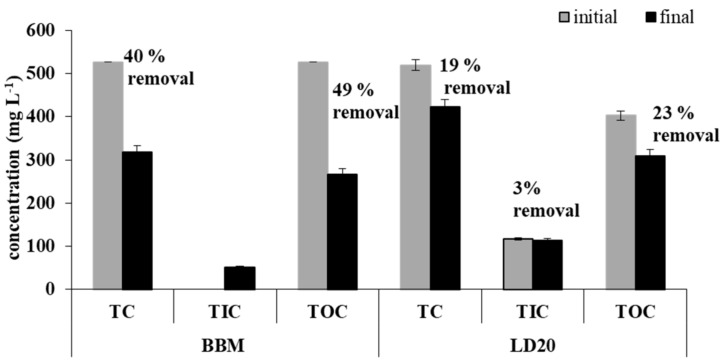
Initial and final concentration as well as the rate of removal of total carbon (TC), total organic carbon (TOC), and total inorganic carbon (TIC) by *A. protothecoides* from BBM medium and liquid digestate (LD 20).

In comparison with the control substrate, the liquid digestate LD 20 exhibited a lower initial total organic carbon (TOC) content and higher initial total inorganic carbon (TIC) content. The content of total carbon of LD 20 was 520 mg L^−1^, with predominance of total organic carbon: 403 mg L^−1^, and the content of total inorganic carbon (TIC) was 117 mg L^−1^ (Figure 3). After the cultivation, there was a 19%, 23%, and 3% decline in the content of TC, TOC, and TIC, respectively (Figure 3). 

### 2.3. Lipid Content and Fatty Acid Profile

The final lipid content was 6.33% in the culture of *A. protothecoides* grown in the control medium and 44.65% in LD 20 (Table 1). LD 20 was characterized by a higher initial C/N ratio (4.6) than the control medium (2.8) (Table 3). In order to further characterize the lipid metabolic profiles, the fatty acid composition was determined with the use of a GC-MS analysis. The composition of fatty acid methyl esters (FAME) is summarized in Table 4. 

**Figure 4:**
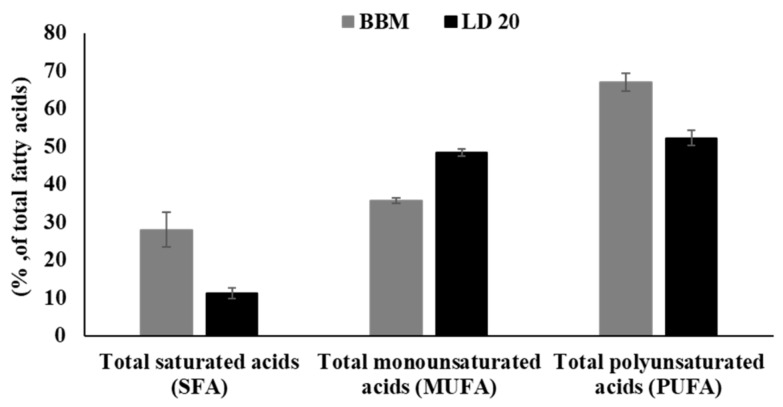
Comparison of the composition of fatty acids (C16–C18) grouped as total saturated acids, total monounsaturated acids, and total polyunsaturated acids. The grey columns represent the control BBM medium and the black columns represent 1:20 liquid digestate (LD 20).

The composition of FAME determined for *A. protothecoides* grown in the control and digestate medium comprised primarily C16-C18 fatty acids: C16:0 (palmitic acid), C18:0 (stearic acid), C18:1 (oleic acid,), C18:2 (linoleic acid), and C18:3 (linolenic acid). The fatty acid composition changed depending on the culture medium used. The fatty acid profile in the biomass of *A. protothecoides* cultured in LD 20 shows the dominance of C18 fatty acids, with the highest content of C18:2 (38.96%) and C18:1 (35.09%). Linolenic acid (34.18%) was the main fatty acid in the culture in the control medium (BBM). *A. protothecoides* cells growing in the liquid digestate were characterized by a significantly lower level of linolenic acid, i.e., 13.31%. The use of liquid digestate as a growth medium changed the content of monounsaturated fatty acids (MUFA) and polyunsaturated fatty acids (PUFA) among C16–C18 (Figure 4).

## 3. Discussion

The present study investigated the effect of liquid digestate used as a nutrient source on the growth parameters and biochemical composition of *A. protothecoides*. Based on the results, it can be concluded that *A. protothceoides* has a capability of adaptation and growth with no inhibition in adequately diluted liquid digestate. The presence of a short lag phase may suggest that the liquid digestate in the dilution of 1:20 is a suitable medium for biomass production. Yu et al. [18] demonstrated that *Chlorella* SDEC-18 cultivated in diluted anaerobically digested effluent kitchen waste did not exhibit a lag phase. Similar results were obtained by Silkina et al. [19] in a study on the utilization of spent anaerobic digestate. The growth of *Nannochloropsis oceanica* and *Scenedesmus quadricuada* strains was characterized by a very short lag adaptation phase [19].

The results obtained in this study indicate that the liquid digestate (LD 20) stimulated a better growth response than the BBM medium in the exponential phase of growth and did not contain sufficient concentrations of inhibitors of *A. protothecoides* growth. Liquid digestate from methane fermentation of maize silage can be used as a substitute growth medium for *A. protothcoides.* In the study conducted by Mayers et al. [20], effluents of anaerobically digested food waste were estimated as a potential nitrogen and a phosphorus source. The results of the studies demonstrate that a suitable dilution of this effluent could meet the nitrogen demands and, partially, the phosphorus demands of *Nannochloropsis* sp. In the study conducted by Massa et al. [21], the specific growth rate in freshwater *Tetradesmus obliquus* and *Botryococcus braunii* and marine *Phaeodactylum tricornutum* cultured in vegetable liquid digestates (composed of corn, rye, and wheat) was 0.12, 0.12, and 0.15 d^−1^, respectively, and was not statistically different from the growth rate noted in cultures grown in standard media. 

Nitrogen can account for 10% or more of the dry weight of algae, depending on the species and N availability for microalgal cells. Its availability is an important determinant of the lipid accumulation process in cells [20]. Liquid digestate fractions are primarily a source of inorganic N (mainly NH_4_) and P-PO_4_ [20]. Regarding the comparison between the digestate and BBM in terms of N removal, it was proven that *A. protothecoides* had the ability to assimilate nitrogen from the liquid digestate. The reduction of the N content in LD results from the process of assimilation of N by *A. protothecoides* cells, which is utilized for formation of proteins and nucleic acids or synthesis of phospholipids [22]. Similar results were obtained by Wang et al. [23], who demonstrated that the total nitrogen content was reduced by 75.7%–82.5% by green microalgae Chlorella *sp* from digested dairy manure. Various publications have shown a range from 7.83% to 97.86% of nitrogen removal by green algae growing in anaerobic digestion effluents: food waste, urban waste, kitchen waste, and agro-industrial wastes [18]. The rate of NH_4_-N removal from the LD 20 by *A. protothecoides* was high, i.e., 93.44% over 12 cultivation days. Massa et al. [21] reported that the values of NH_4_-N removal efficiency in *T. obliques* and *B. braunii* cultures grown in liquid digestates obtained from anaerobic digestion of vegetable biomass were 99.2% and 88.5%, respectively. Ammonia is the preferred nitrogen form for microalgae, as ammonia uptake into the cell is less energy consuming in comparison with other nitrogen sources [24]. 

Phosphorus is an important micronutrient indispensable for microalgal growth, although its content in algal biomass ranges from 0.05% to 3.3% [25]. It is the main component of nucleic acids, ATP, and phospholipids. Algae assimilate phosphorus in the orthophosphate form, but other forms, i.e., mainly dissolved organic phosphorus and insoluble phosphorus compounds, are used by algae as well [24]. An insufficient phosphorus concentration in the medium can lead to a reduction in the rate of cell divisions [18]. Effluents from anaerobic digestion exhibit a varied phosphorus level. For instance, autoclaved and diluted (50%) digestate from anaerobic digestion of food waste contained 136 mg L^−1^ of phosphorus [20], undiluted agro-industrial waste: 114mg L^−1^ and 130 mg L^−1^ [26], cattle manure: 1119 mg L^−1^ [27], and effluent of livestock waste: 140 mg L^−1^ and 75 mg L^−1^ in raw and pretreated wastewater, respectively [28]. The differences in the phosphorus content may result from the variation of the substrate, which is subjected to the methane fermentation process, and from the mode of wastewater pretreatment. A low level of P was reported by reported Yu et al. [18] in a study where digested effluent from kitchen waste contained 0.95 mg L^−1^ of the element. Liquid digestate that is obtained by mechanical separation of raw digestate is characterized by a low P content and a high K content [29]. The liquid digestate used in this study was characterized by a low P content. In this study, the phosphorus removal rates in LD 20 and BBM were 78.4% (1.63 mg L^−1^) and 4% (2.23 mg L^−1^), respectively (Table 3). Wang et al. [23] observed a reduction of total phosphorus ranging from 62.5% to 74.7% in differently diluted anaerobic digested dairy manure (10, 15, 20, and 25 dilution). In recent investigations conducted with the use of anaerobically digested effluent from kitchen waste, it was demonstrated that the efficiency of total phosphorus (TP) removal in four species of microalgae (*Scenedesmus* SDEC-8, *Scenedesmus* SDEC-13, *Monoraphidium* SDEC-17, *Chlorella* SDEC-18) ranged from 78.53% to 96.23% at the initial TP concentration of 28.14 ± 0.11 mg L^−1^ [18]. 

Carbon is one of the most important elements utilized by algae and constituted 46%–50 % of dry microalgal biomass [22]. In the process of methane fermentation, the organic fraction of wastes is converted to CH_4_ and CO_2_, which is dissolved into a liquid and generates a bicarbonate-carbonate buffer of an anaerobic liquid [30]. Xia and Murphy [14] concluded that the content of carbon sources in digestate may be much lower than that required for microalgal growth, and external inorganic sources should be supplied to support microalgal growth. To prevent the depletion of the carbon source in the substrate, the experimental *A. protothecoides* cultures were continuously aerated with sterile air. As reported by Sforza et al. [31] in a study of mixotrophic growth of *Chlorella protothecoides*, CO_2_ excess can block the metabolism of organic carbon substrates. Literature data indicate that the C/N ratio is an important determinant of nitrogen and phosphate removal efficiency and microalgal growth [24]. The initial C/N ratio was 4.6 in LD and 2.8 in BBM (Table 3). The data on the removal of micro- and macroelements prove that *A. protothecoides* algae have a high potential to reduce nutrients in the digestate. 

The main factors influencing lipid accumulation in algal cells include light intensity, carbon source, temperature, content of macro- and micro-nutrients, e.g., nitrogen, and the concentration of organic matter in the case of wastewater [32]. The lipid content in cells growing on the waste medium (LD 20) was 7-fold higher than the value in the cells growing in the control medium. Phosphorus availability is one of the determinants of lipid biosynthesis [33]. The diluted liquid digestate was characterized by a low level of phosphorus, which may have additionally influenced the accumulation of lipids in *A*. *protothecoides* as a stress factor. These results indicate that the digestate medium stimulated *A. protothecoides* cells to accumulate lipids. Investigations reported by Liang et al. [33] demonstrated the maximum lipid content in *Chlorella* sp. in low phosphorus conditions. Similar results of the lipid content in phosphorus limitation conditions (in anaerobically digested effluent from kitchen waste) were reported by Yu et al. [18], who observed an approximately 70% increase in the lipid content in two analyzed strains (*Scenedesmus* SDEC-8 and *Chlorella* SDEC-18) in comparison with a culture on BG 11 medium. Similar results were obtained by Massa et al. [22], i.e., an increase in the lipid content in the biomass of *Tetradesmus obliquus* cultured in vegetable liquid digestate, compared with Zarrouk media.

The fatty acid profile influences fuel properties. In this study, the composition of *A. protothecoides* fatty acids shows that C16-C18 acids with no more than three degrees of unsaturation, which are the determinants of the quality of biodiesel, are the dominant fatty acids. The requirements for fatty acid methyl esters intended for use as biofuels specified in the PN-EN 14,214 standard comprise a reduction of the linolenic acid content below 12% [4]. It is interesting to note that the percentage content of oleic acid C18:1 was elevated from 2.93% in the control cells to 35.25% of the total composition in *A. protothecoides* cultivated in the liquid digestate. Oleic acid and palmitic acids are one of the most desirable fatty acids for the production of biodiesel [26]. In a study reported by Shin et al. [34], oleic acid dominated among all fatty acids produced by *Scenedesmus bijuga* cultivated in 1/10, 1/20, and 1/30 diluted anaerobically digested food wastewater effluent. Similarly, investigations conducted by Massa et al. [21] demonstrated that the fatty acid profile in *T. obliquus* cultivated on diluted vegetable liquid digestate was characterized by a high content of oleic acid, i.e., 31.2%. 

The results show an increase in the MUFA level in *A*. *protothecoides* cells from 35.64% in the control medium to 48.40% in the liquid digestate. Additionally, a reduction of the PUFA content in *A. protothecoides* from 67.02% in the BBM medium to 52.27% in the liquid digestate was recorded. Triacylglycerols (TAG), which are regarded as a good source for the production of biofuels, are mainly composed of monounsaturated and saturated fatty acids [35]. TAG biosynthesis is usually increased in conditions that are stressful to microalgae. Hence, the diluted liquid digestate, exhibiting low contents of P and N, provides favorable conditions for the accumulation of lipids and TAG in *A. protothecoides* cells. Similar results of reduced polyunsaturated fatty acids in *T*. *obliquus* cultivated on zootechnical waste and vegetable waste were reported by Massa et al. [21] in their study of the application of anaerobic digestates as growth media. Due to their susceptibility to oxidation, polyunsaturated fatty acids are not a suitable substrate for biodiesel production. Oxidative stability is a key factor in the process of storage of methyl ester biodiesel. The oxidative stability of FAME declines with increasing levels of unsaturation; therefore, limitation of their proportion in the final oil product is advisable [20,36].

## 4. Materials and Methods

### 4.1. Culture Conditions

*A. protothecoides* was obtained from the SAG Culture Collection (211-7b). The *A. protothecoides* inoculum was cultured in sterile liquid BBM medium enriched with peptone (0.1% *w/v*). 

Liquid digestate (LD) collected after methane fermentation of maize silage was used as a medium for *A. protothecoides* cultivation. After sedimentation, the digestate was centrifuged, filtered through glass microfiber filters, and sterilized in an autoclave. The sterilized digestate was diluted 10 and 20 times (LD 10, LD 20, respectively) with deionized water to increase the transmission of light. BBM medium (Table 5) with peptone (0.1% *w*/*v*) was used as a control in the experiment [37]. The peptone-supplemented BBM medium is used as a substrate in investigations of lipid accumulation in *A. protothecoides* [31]. The growth experiments were performed in 500 mL Erlenmeyer flasks with 200 mL of the medium. The samples were continuously aerated with sterile air at 12 L·h^−1^ airflow and shaken at 100 rpm. The cultivation conditions included light intensity under 80 µmol photons m^−2^ s^−1^ in dark/light cycles of 18 h light/6 h dark. Each culture variant was performed in three independent biological replicates.

### 4.2. Physico-Chemical Analysis of The Digestate

The physicochemical properties of the digestate such as total solids (TS), volatile solids (VS), ash, and total nitrogen were analyzed following the procedure described by Oleszek et al. [38]. Additionally, the chemical oxygen demand (COD) and biological oxygen demand (BOD) were determined. COD was tested spectrophotometrically with Hach Lange tests in accordance with the manufacturer’s protocol. The manometric respirometric test of BOD was carried out with the OxiTop Control system. The test is based on automatic measurement of pressure reduction in a closed bottle at a temperature of 25 °C. The decline in the pressure is associated with the fact that a certain amount of oxygen is consumed during the process of organic matter biodegradation and the CO_2_ formed is removed from the gas space by means of a reaction with granulated NaOH.

The N content was analyzed with the Kjeldahl method. The content of NH_4_-N was determined with the distillation method using a Behr steam distillation unit S4. The N-NO_3_^−^ and N-NO_2_^−^ contents were determined with the colorimetric method using the Nitrate Test and Nitrite Test from Merck in accordance with the manufacturer’s protocol. The content of N_org._ was calculated by subtraction of inorganic nitrogen (NH_4_-N, N-NO_3_^−^ and N-NO_2_^−^) from N content.

The content of total carbon, total inorganic carbon, and total organic carbon was determined spectrophotometrically using Hach Lange tests in accordance with the manufacturer’s protocol.

The content of macro and microelements was analyzed with an Inductive Coupled Plasma-Optical Emission Spectrometer (ICP-OES) according to the method described previously by Oleszek et al. [19]. 1-mL samples of the tested media were mineralized using a microwave mineralizer Berghoff Speedwave Four in teflon vessels DAP 100 in a mixture of concentrated acids: HNO_3_, HCl, and HF in the proportion of 9:3:1. Next, the solutions obtained were analyzed with ICP-OES (Thermo Scientific iCAP Series 6500) equipped with a Charge Injection Device (CID) detector and TEVA software. The ICP OES operational parameters were as follows: auxiliary flow of 0.4 L min^−1^, carrier gas flow rate of 0.65 L min^−1^, coolant gas flow rate 16 L min^−1^, RF power of 1150 W, frequency of RF generator of 27.12 MHz, pump rate of 50 rpm, and axial viewing configuration. Two multi-element solutions from Inorganic Ventures (Virginia, US) were used as standards. 

### 4.3. Determination of Algal Growth 

The growth of *A. protothecoides* was measured by the number of cells monitored using the Bürker chamber under an OLYMPUS/CKX41SF microscope. The specific growth rate was calculated in the exponential phase using the equation:

µ = ln (N_2_/N_1_)/(t_2_ − t_1_) (1)

where µ is the specific growth rate, and N_1_ and N_2_ are the numbers of cells at the start (t_1_) and the end of the exponential growth phase (t_2_).

### 4.4. Lipid Extraction

Lipids were extracted from centrifuged *A. protothecoides* cells after 11 days of cultivation. The analysis of the total lipid content was conducted with the modified Bligh and Dyer method [39] preceded by disruption of the cells by sonication (Sonics vibra cell 500) and gravimetric determination of total lipids. Briefly, the analyses consisted of the following steps: chloroform and methanol were added at a ratio of 1:2 (*v*/*v*) to the centrifuged algal biomass (with determined dry weight) and the samples were shaken for 20 min on a shaker; next, chloroform was added and the samples were shaken on a vortex. This was followed by adding water and shaking. The samples were centrifuged for 10 min at 2000 rpm; next, the lower layers of the samples were collected and the solvents were removed by evaporation (vacuum evaporator).

### 4.5. Analysis of Fatty Acids

The fatty acid profile was determined after the cultivation process. The analysis was carried out with the use of gas chromatography as described by Krzemińska et al. [40]. Briefly, the analysis of the fatty acid composition consisted of the following steps: 1 mL of crude oil was suspended in 1 mL of HPLC grade hexane. Next, it was mixed with 6 mL of 0.5 M KOH in HPLC grade methanol and hydrolyzed at 80 °C for 1 h. Subsequently, 6 mL of 10% BF_3_ in 100% methanol (Sigma Aldrich) were added. The esterification procedure was performed at 100 °C for 20 min. After cooling, 1 mL of HPLC grade hexane and 10 mL of a saturated NaCl solution were added. The fatty acid methyl esters (FAME) resulting from the esterification step accumulated in the hexane layer. The samples were analyzed on a Trace GC Ultra with chromatograph coupled with ion trap mass spectrometer ITQ 1100 (Thermo Scientific, Madison, WI, USA) using a 105 m Rtx-2330 column with I.D. of 0.25 mm and 0.25 µm film thickness (Restek). Helium at a flow rate of 2.4 mL/min was the carrier gas. The components were identified based on a previous analysis of the mixture of standard solutions from Supelco® 37 Component FAME Mix solutions (Sigma, Supelco, Saint-Louis, MI, USA).

The results are presented as the means of nine measurements from three biological replicates. The significance of the differences in the measured parameters was compared using one-way or two-way ANOVA followed by a Tukey HSD test (STATISTICA 12).

## 5. Conclusions

The results indicate that the use of sterilized diluted liquid digestate from methane fermentation of maize silage stimulated *A. protothecoides* growth and the accumulation of lipids. In the liquid digestate, *A. protothecoides* removed 79.4% of nitrogen and 76.4% of phosphorus from the substrate. A higher level of monounsaturated fatty acids compared to the control medium was noted in the cells of microalgae growing in the liquid digestate. The increase in the content of oleic acid and the decline in the level of linolenic acid in *A. protothecoides* cells in the liquid digestate is a beneficial change in the fatty acid profile in terms of the use of lipids for biodiesel production. Since maize silage is the main feedstock used in European biogas plants, the use of the liquid digestate from methane fermentation of maize silage as a substrate for biomass production is one of the ways to optimize the bioprocess of microalgal biomass production and to reduce nutrients in the liquid digestate.

## Figures and Tables

**Table 1:** Effects of the substrate on the specific growth rate, doubling time, and lipid content in *A. protothecoides*. Data are means (± SDs) of nine measurements.

Medium	Specific Growth Rate 0–6 (day ^−1^)	Doubling Time 0–6 (h)	Specific Growth Rate 7–11 (day ^−1^)	Doubling Time 7–11 (h)	Lipid Content (% of Dry Weight) *
BBM	0.302 ± 0.056	51.573 ± 7.621	0.675 ± 0.039	24.71 ± 1.45	6.33 ± 1.4 *a*
LD 20	0.412 ± 0.044	40.767 ± 4.231	0.704 ± 0.053	23.72 ± 3.24	44.65 ± 2.65 *b*

* Different letters (a,b) indicate significant differences *p* < 0.01.

**Table 2:** Physicochemical parameters of the tested anaerobic digestate.

Parameters	Unit	Mean ± SD
TS	%	3.64 ± 0.12
VS	% TS	59.30 ± 1.55
Ash	% TS	40.70 ± 1.55
BOD	mg O_2_ L^−1^	3985 ± 156
COD	mg O_2_ L^−1^	9140 ± 90
BOD/COD	-	0.44 ± 0.032

TS—total solids, vs.—volatile solids, BOD—biological oxygen demand, COD—chemical oxygen demand.

**Table 3:** Removal of macro and microelements by *A. protothecoides* from the liquid digestate (LD 20) and BBM medium.

Removal of Element		P	K	Na	Zn	Mg	Mn	Mo	Fe	Co	Cu	N-NO_2_^−^	N-NO_3_^−^	C/N
	unit	LD 20												
Initial concentration	mg L^−1^	2.08 ± 0.01 a	192.49 ± 2.67 a	25.13 ± 0.00 a	0.57 ± 0.02 a	2.61 ± 0.21 a	1.97 ± 0.01 a	0.21 ± 0.00 a	1.79 ± 0.04 a	0.00 ± 0.00 a	0.03 ± 0.00 a	0.25 ± 0.01 a	4.15	4.6
Final concentration	mg L^−1^	0.45 ± 0.00 b	174.21 ± 0.54 b	16.97 ± 0.28 b	0.21 ± 0.08 a	0.61 ± 0.18 b	1.85 ± 0.03 b	0.08 ± 0.00 b	1.03 ± 0.02 b	0.00 ± 0.00 a	0.02 ± 0.00 a	0.15 ± 0.01 b	1.20	17.8
Removal	mg L^−1^	1.63	18.28	8.16	0.36	2.00	0.12	0.13	0.76	0.00	0.01	0.11	2.95	
Removal	%	78.4	9.5	32.5	63.1	76.7	5.9	61.2	42.2	-	32.6	43.1	71.1	
		BBM												
Initial concentration	mg L^−1^	53.3 ± 0.00 c	105.80 ± 0.00 c	77.60 ± 0.00 c	2.00 ± 0.00 b	7.40 ± 0.00 c	0.40 ± 0.00 c	0.47 ± 0.00 c	1.00 ± 0.01 b	0,10 ± 0.00 b	0.63 ± 0.01 b	nd	45.05	2.8
Final concentration	mg L^−1^	51.07 ± 1.04 d	83.20 ± 0.07 d	69.29 ± 1.33 d	1.43 ± 0.28 c	6.66 ± 0.43 d	0.16 ± 0.02 d	0.42 ± 0.01 d	0.00 ± 0.00 c	0.09 ± 0.02 b	0.52 ± 0.00 c	nd	0.00	12.7
Removal	mg L^−1^	2.23	22.60	8.31	0.57	0.74	0.24	0.05	1.00	0.01	0.11		45.05	
Removal	%	4.0	21.4	10.7	28.7	10.0	60.1	10.0	100.0	8.7	17.5		100	

Means with different letters (a–d) in the column differ significantly in post-hoc Tukey test at *p* < 0.05; nd—not detected.

**Table 4:** Fatty acid composition of *A. protothecoides*.

	Medium	
Distribution of fatty acids (%, of total fatty acids) *	BBM	LD 1:20
16:0 (Palmitic acid)	14.92 ± 1.88	9.46 ± 1.10
18:0 (Stearic acid)	13.13 ± 2.73	1.76 ± 0.28
18:1 (Oleic acid)	2.93 ± 0.34	35.09 ± 0.08
18:2 (Linoleic acid)	32.85 ± 1.05	38.96 ± 1.05
18:3(Linolenic acid)	34.18 ± 1.31	13.31 ± 0.95
C16-C18	98.00	98.56

* fatty acids among total C16-C18. Data are means (± SDs) of 6 measurements.

**Table 5:** Composition of of Bold Basal Medium.

Component	Stock Solution g L^−1^		Quantity Used (mL L^−1^)
NaNO_3_	25		10
CaCl_2_·2H_2_O	2.50		10
MgSO_4_·7H_2_O	7.50		10
K_2_HPO_4_	7.50		10
KH_2_PO_4_	17.50		10
NaCl	2.50		10
EDTA solution g L^−1^			
EDTA	50.00		1 mL
KOH	31		
Acidified Iron Solution (to 100 mL)			
FeSO_4_·7H_2_O	0.498 g		1 mL
H_2_SO_4_ (96%)	0.1 mL		
Trace metals solution (g L^−1^)			
ZnSO_4_·7H_2_O	8.82		1 mL
MnCl_2_·4H_2_O	1.44		
MoO_3_	0.71		
CuSO_4_·5H_2_O	1.57		
Co(NO_3_)_2_·6H_2_O	0.49		
